# A rise in the frequency of *lasR* mutant *Pseudomonas aeruginosa* among keratitis isolates between 1993 and 2021

**DOI:** 10.3389/fcimb.2023.1286842

**Published:** 2023-10-31

**Authors:** Robert M. Q. Shanks, Sarah Atta, Nicholas A. Stella, Chollapadi V. Sundar-Raj, John E. Romanowski, Arman S. Grewal, Hazel Q. Shanks, Sonya M. Mumper, Deepinder K. Dhaliwal, Alex Mammen, Jake D. Callaghan, Rachel C. Calvario, Eric G. Romanowski, Regis P. Kowalski, Michael E. Zegans, Vishal Jhanji

**Affiliations:** ^1^ Charles T. Campbell Laboratory of Ophthalmic Microbiology, Department of Ophthalmology, University of Pittsburgh School of Medicine, Pittsburgh, PA, United States; ^2^ Department of Surgery, Geisel School of Medicine at Dartmouth, Hanover, NH, United States; ^3^ Department of Microbiology and Immunology, Geisel School of Medicine at Dartmouth, Hanover, NH, United States

**Keywords:** keratitis, *Pseudomonas aeruginosa*, ocular infection, quorum sensing, transcription factor, protease

## Abstract

**Introduction:**

*Pseudomonas aeruginosa* causes vision threatening keratitis. The *LasR* transcription factor regulates virulence factors in response to the quorum sensing molecule N-3-oxo-dodecanoyl-L-homoserine lactone. *P. aeruginosa* isolates with *lasR* mutations are characterized by an iridescent high sheen phenotype caused by a build-up of 2-heptyl-4-quinolone. A previous study demonstrated 22% (n=101) of *P. aeruginosa* keratitis isolates from India between 2010 and 2016 were sheen positive *lasR* mutants, and the sheen phenotype correlated with worse clinical outcomes for patients. In this study, a longitudinal collection of *P. aeruginosa* keratitis isolates from Eastern North America were screened for *lasR* mutations by the sheen phenotype and sequencing of the *lasR* gene.

**Methods:**

Keratitis isolates (n=399) were classified by sheen phenotype. The *lasR* gene was cloned from a subset of isolates, sequenced, and tested for loss of function or dominant-negative status based on an azocasein protease assay. A retrospective chart review compared outcomes of keratitis patients infected by sheen positive and negative isolates.

**Results:**

A significant increase in sheen positive isolates was observed between 1993 and 2021. Extracellular protease activity was reduced among the sheen positive isolates and a defined *lasR* mutant. Cloned *lasR* alleles from the sheen positive isolates were loss of function or dominant negative and differed in sequence from previously reported ocular *lasR* mutant alleles. Retrospective analysis of patient information suggested significantly better visual outcomes for patients infected by sheen positive isolates.

**Discussion:**

These results indicate an increase in *lasR* mutations among keratitis isolates in the United States and suggest that endemic *lasR* mutants can cause keratitis.

## Introduction

The bacterium *Pseudomonas aeruginosa* is the most frequent cause of contact lens associated microbial keratitis and is of concern because keratitis caused by *P. aeruginosa* has rapid progression and poor clinical outcomes (Shen et al., 2014; Vazirani et al., 2015). *P. aeruginosa* keratitis isolates resistant to fluoroquinolones and other antibiotics typically used to treat keratitis have been reported (Alexandrakis et al., 2000; Rhee et al., 2004; Bispo et al., 2022; Khan et al., 2023). Microbial keratitis caused by antibiotic resistant *P. aeruginosa* isolates correlates with worse clinical outcomes including an increase in corneal perforations from 12% of cases with normal *P. aeruginosa* to 52% with multidrug-resistant (MDR) *P. aeruginosa* (Vazirani et al., 2015; Fernandes et al., 2016). Beyond resistance, *P. aeruginosa* has numerous virulence factors associated with establishing corneal infections (Pearlman et al., 2013; Hilliam et al., 2020). These include pathogen-associated molecular pattern (PAMP) such as lipopolysaccharide and flagellin (Fleiszig and Evans, 2002; Willcox, 2007), a variety of proteases including elastases (LasA and LasB) and *Pseudomonas aeruginosa* Small Protease (PASP) (O'Callaghan et al., 2019; Hilliam et al., 2020), and the type III secretion system that are important for virulence in experimental models (Zhu et al., 2006; Evans and Fleiszig, 2013; Pearlman et al., 2013; Hilliam et al., 2020). These virulence factors are highly regulated through multiple transcriptional regulators including the LasR quorum sensing master regulator that mediates population density collective responses (Lee and Zhang, 2015; Moradali et al., 2017). Likely the most studied among these in *P. aeruginosa* is the LasR transcription factor. LasR responds to quorum sensing molecule N-(3-oxododecanoyl) homoserine lactone, controls a large portion of the *P. aeruginosa* genome, and is an important regulator of pathogenesis in lung and burn infection models (Rumbaugh et al., 1999; Pearson et al., 2000; Lesprit et al., 2003), as it positively regulates a number of pro-virulence factors including elastase proteases LasA and LasB, and rhamnolipids (Kariminik et al., 2017; Moradali et al., 2017).

A prior study on the keratitis isolates of *P. aeruginosa* from the Steroids for Corneal Ulcers Trial (SCUT) (Hammond et al., 2016) reported a colony iridescent sheen positive phenotype in 22 of the 101 isolates taken during the course of the study from India (Hammond et al., 2016). This sheen phenotype correlated with significantly worse visual outcomes. These included significantly reduced visual acuity and infiltrate/scar size for patients infected sheen isolates compared to typical *P. aeruginosa* (Hammond et al., 2016). The basis for the sheen phenotype has been shown to be due to mutation of the *lasR* transcription factor gene (D'Argenio et al., 2007). LasR is a positive regulator of the gene *pqsH*, which codes for an enzyme that converts 2-heptyl-4-quinolone (HHQ) to heptyl-3-hydroxy-4(1H)-quinone (PQS) (D'Argenio et al., 2007). In the absence of LasR function, HHQ builds up in the cell and creates the sheen phenotype. PQS is an important signaling molecule known as *Pseudomonas* quinolone signal (Garcia-Reyes et al., 2020). Surprisingly only two nonsynonymous mutations in the *lasR* gene were detected in 21 out of 22 sequenced sheen positive isolates suggesting that the mutations were already present in strains endemic to the country (India). By contrast, in chronic lung infections, such as those associated with cystic fibrosis, *P. aeruginosa* are frequently observed to gain mutations in *lasR* (Smith et al., 2006; D'Argenio et al., 2007). In the airway, patients are thought to be initially infected by wild-type (WT) *P. aeruginosa*, and *lasR* mutants can then increase over time (Kohler et al., 2009). Like keratitis patients, cystic fibrosis patients infected with *lasR* mutants have been recorded to experience worse disease progression compared to patients infected by wild-type *P. aeruginosa* (Hoffman et al., 2009). Together these prior studies suggest that sheen isolates are associated with worse clinical outcomes.

In the SCUT study, all of the sheen isolates tested were isolated between 2006-2010 and caused largely by isolates with one of two *lasR* mutations (Hammond et al., 2016). This study sought to determine whether sheen isolates were a general phenomenon among *P. aeruginosa* keratitis isolates or a geographically isolated observation and, if wide-spread, whether the same mutant alleles of *lasR* were present and associated with worse visual outcomes. Here we found a concerning increase in *lasR* mutants among the keratitis isolates taken in a tertiary care hospital in the Eastern United States. Data suggests that the mutations were highly variable with one exception, being an insertion element present in several strains; moreover, retrospective analysis suggests that patients with *lasR* mutations had better visual outcomes contrary to the former study.

## Materials and methods

### Microbiology

De-identified *P. aeruginosa* strains isolated from the corneas of patients with keratitis were retrieved from a clinical tissue bank which is used for validation of diagnostic testing and antibiotic evaluation. The *P. aeruginosa* isolates were collected from 1993 through 2021 by The Charles T. Campbell Ophthalmic Microbiology Laboratory at the University of Pittsburgh School of Medicine and stored at -80°C. *P. aeruginosa* isolates were plated on tryptic soy agar with 5% sheep’s red blood cells and incubated for 18-20 hours at 37°C and sheen phenotype was established visually.

### Molecular biology

The *lasR* gene was deleted from strain PaC (Kowalski et al., 2001) using allelic exchange with plasmid pMQ30, as previously described (Shanks et al., 2006). The plasmid was modified with a Δ*lasR* allele cloned from PA14 Δ*lasR* (Hammond et al., 2016) to generate pMQ767. Primers to amplify the *lasR* region (approximately 500 bp upstream and downstream of the ORF) were 4835 and 4836 and listed in **Table 1**. The resultant strain was verified by PCR and whole genome sequencing. The *lasR* ORF was amplified by PCR and cloned into shuttle vector pMQ132 under control of the *Escherichia coli lac* promoter using yeast homologous recombination as previously described (Shanks et al., 2009). Primers to amplify the *lasR* ORF were 3217 and 3218 (**Table 1**). Plasmids were sequenced at the University of Pittsburgh Genomics Core or PlasmidSaurus.

**Table 1:** Oligonucleotide primers used in this study.

Primer name	Primer sequence (5’ to 3’)
3217 *lasR* cloning primer	ggataacaatttcacacaggaaacagctATGGCCTTGGTTGACGGTTTTCTTG
3218 *lasR* cloning primer	atctgtatcaggctgaaaatcttctctcatccgccaaaGGAAAGCCAGGAAACTTTCTGG
4835 primer to clone *lasR* deletion allele	cgacgttgtaaaacgacggccagtgccaagcttgcatgccGCTCGGCCTGTTCTGTGTCG
4836 primer to clone *lasR* deletion allele	ggaaacagctatgaccatgattacgaattcgagctcGAGTGCGTCATAACCATCGATTTC

Uppercase letters direct priming and lowercase letter direct homologous recombination.

### Protease assays

Milk agar plates were made with brain heart infusion and skim milk as previously described (Sokol et al., 1979). Bacteria were incubated at 37°C for 24 hours and zones of clearing were measured on at least three separate occasions. For more quantitative analysis azocasein was used as previously described (Shanks et al., 2013).

### Antibiotic susceptibility testing

The minimum inhibitory concentrations (MICs) of *Pseudomonas aeruginosa* keratitis isolates were determined to ciprofloxacin (CIP), tobramycin (TOB), and ceftazidime (CAZ) using MIC strips (Fisher Scientific, LIOFILCHEM, MA) on Mueller-Hinton II agar (BD BBL) agar as previously described (Harshaw et al., 2021). The keratitis isolates tested, taken from the cornea, were chosen arbitrarily out of the deidentified strain bank. The isolates in question were collected anonymously from 1993 to 2021 by the Charles T. Campbell Ophthalmic Microbiology Laboratory and stored at -80°C. The antibiotic susceptibility was determined by comparing the MIC of each to the Clinical and Laboratory Standards Institute breakpoints (Humphries et al., 2021).

### Chart review

Retrospective review of medical records of all patients diagnosed with culture-positive *P. aeruginosa* keratitis at the University of Pittsburgh Medical Center between 2017 and 2021 was performed. The study was approved by the Institutional Review Board of the University of Pittsburgh and followed the tenets of the Declaration of Helsinki. Clinical data were collected for each patient, including clinical features, treatment, and outcomes. Demographic features were recorded, including gender and age. Visual acuity was recorded at presentation and after resolution. The visual outcomes were BCVA on the Snellen chart and converted to LogMAR.

### Statistical analysis

Graph-pad Prism was used to perform Mann-Whitney and ANOVA analysis with Tukey’s post-test, chi-square, and Fisher’s exact tests. P<0.05 was considered significant.

## Results

### An increase in sheen positive *Pseudomonas aeruginosa* was observed among keratitis isolates from a North Eastern United States tertiary care facility between 1993-2021

**Figure 1:**
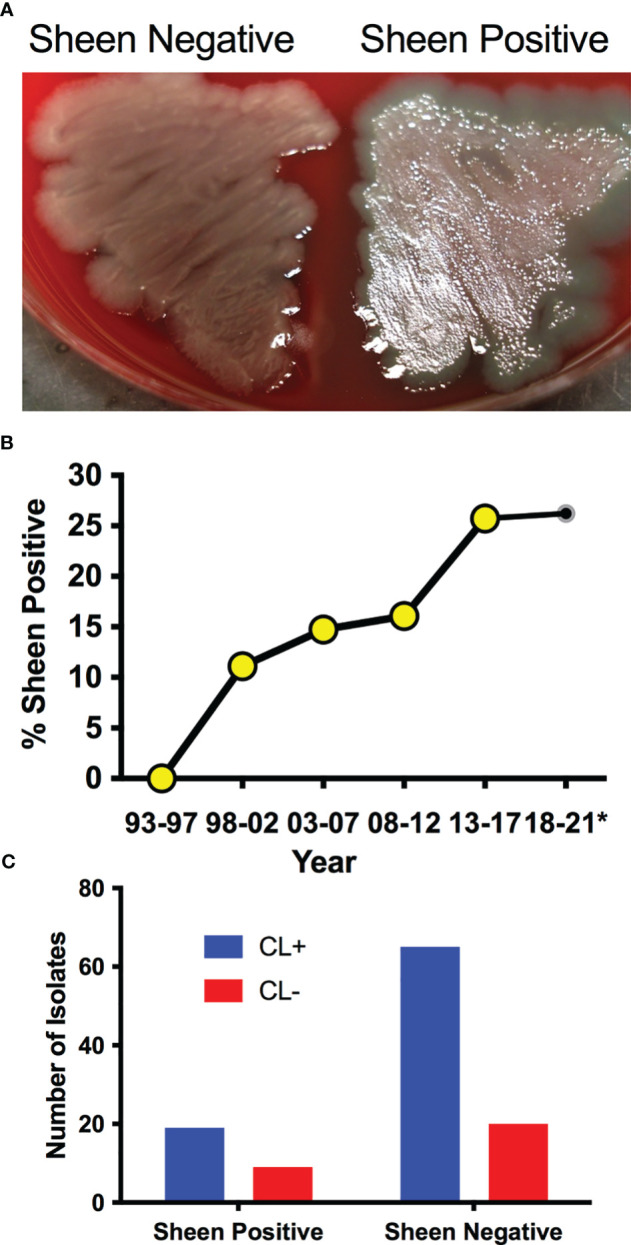
*P. aeruginosa* keratitis isolates with a sheen phenotype are increasing over the last two decades. **(A)** Appearance of sheen negative and positive *P. aeruginosa* keratitis isolates on blood agar. **(B)** Frequency of PA keratitis isolates with sheen positive phenotype. 5 year periods are shown except for 2018-2021 as denoted by the asterisk. n=399, n≥50 per time period. **(C)** Correlation between contact lens (CL) use and sheen status. p=0.455.

*P. aeruginosa* keratitis isolates between 1993-2021 were evaluated for sheen phenotypes on blood agar plates (**Figure 1A**). The isolate collection and storage approach remained consistent over that time period. A notable increase in sheen positive isolates was observed over the time frame going from 0% between 1993-1997 to 26.2% between 2018-2021 (**Figure 1B**). All time frames tested were significantly different from 1993-1997 by Fisher’s Exact and chi-square Test and (p<0.01).

Contact lens use is the major risk factor for *P. aeruginosa* keratitis (Green et al., 2019; Enzor et al., 2021; Ung and Chodosh, 2021). Where possible, the contact lens use status of the patient was correlated with the sheen phenotype and no significant difference was observed (p=0.45 by Fisher’s Exact Test) with 23% sheen positive isolates from contact lens associated keratitis (19/84) and 31% sheen positive isolates from non-contact lens associated keratitis (9/29) (**Figure 1C**).

We also screened a small library of fluoroquinolone resistant isolates from keratitis patients in the New York City area obtained before 2001 (Kowalski et al., 2001) and found that two out of six where sheen positive (33.3%), further suggesting that sheen positive ocular clinical isolates are a general rather than geographically isolated phenomenon.

### Sequence analysis indicates a variety of mutations and was suggestive of one endemic strain

The *lasR* allele from a subset of arbitrarily chosen strains were cloned into a shuttle vector and sequenced. The prior study with patients from India reported multiple independent isolates with two specific LasR variants I215S (14/22) and P117L (7/22), and one having a missense mutation yielding V221L (1/22) (Hammond et al., 2016). By contrast, our study did not find these mutations and found a wider variety of alterations in the *lasR* sequence. We cloned and sequenced the *lasR* open reading frame (ORF) from 27 strains including 4 from sheen negative isolates and 23 from sheen positive isolates (**Table 2**). Of the sheen positive strains, 1 had a wild-type *lasR* sequence, others had amino acid substitutions, deletions of insertions that created out of frame mutations, premature stop codons, and insertion elements. Strikingly, 4 of 6 with insertion elements had identical insertions at base pair 126 despite being found over 20 years.

**Table 2:** Sequence analysis of PA *lasR* gene (717 bp long).

Isolate	Sheen Phenotype	*lasR* Allele (relative to WT)
K129	Positive	insertion element ISPst7 at bp 126 (isolated 1991)
K828	Positive	deletion after AA 115, frame shift
K846	Positive	truncation after S44
K944	Positive	N55Y
K1001	Positive	N55Y L148P
K1093	Positive	P149S G191D
K1255	Positive	S219F
K1322	Positive	insertion element ISPst7 at bp 126 (isolated 2001)
K1471	Positive	R180W
K1494	Positive	insertion element ISPst7 at bp 126 (isolated 2003)
K1697	Positive	insertion after bp 254, frame shift
K1713	Positive	A105T
K2204	Negative	WT^a^
K2333	Positive	R180Q
K2361	Positive	WT
K2386	Positive	truncation after L151
K2523	Positive	IS5 family insertion element at bp 126
K2634	Positive	frame shift after P85
K2740	Positive	A166G
K2942	Positive	T193I
K2961	Positive	IS630 family Insertion element at bp 599
K2970	Positive	I200F
K2979	Positive	insertion element ISPst7 at bp 126 (isolated 2017)
PAB	Positive	WT
PAC	Negative	WT
PAD	Positive	deletion of 13 base pairs at bp 113, frame shift
PAO1	Negative	WT
PA14	Negative	WT

^a^WT indicates no change from the PAO1/PA14 amino acid sequence.

### Sheen positive keratitis isolates had increased susceptibility rates to a ceftazidime

It has been reported that *lasR* isolates have altered susceptibility to a variety of antibiotics (Hoffman et al., 2010; Qi et al., 2016; Abisado-Duque et al., 2023). To test whether sheen status had an impact on susceptibility to the major topical antibiotics for *P. aeruginosa* keratitis (Lin et al., 2019), we evaluated minimum inhibitory concentrations from keratitis isolates (**Table 3**). Drugs from three different antibiotic classes were evaluated. There was no difference in the percent susceptible to the fluoroquinolone ciprofloxacin for sheen positive versus negative isolates (92.5% compared to 93.5% respectively, p=0.29 Fisher’s Exact test). For the aminoglycoside tobramycin, there was a non-significant trend toward a higher percentage of susceptible isolates in the sheen negative group which was 10% higher than the sheen positive group (p=0.23). While the cephalosporin ceftazidime is not used as commonly for keratitis, it has been used successfully used to treat keratitis and has been suggested as an alternative for treatment of aminoglycoside and fluoroquinolone resistant isolates (Rhee et al., 2004; Dehghani et al., 2009; Subedi et al., 2018). For ceftazidime, the sheen negative isolates had a higher frequency of susceptibility than the sheen positive isolates (98.1% vs 85.3% respectively, p=0.01).

**Table 3:** Descriptive statistics of minimum inhibitory concentrations (MICs) for *Pseudomonas aeruginosa* keratitis isolates to ceftazadime, ciprofloxacin, and tobramycin.

	N	Median (µg/ml)	Mode (µg/ml)	MIC_50_ (µg/ml)	MIC_90_ (µg/ml)	Range	Susceptibility (%)
Sheen +							
ceftazidime	34	2	1.5	2	8	0.75 - >256	85.3
ciprofloxacin	41	0.19	0.19	0.19	0.5	0.064 - 1	92.5
tobramycin	41	1.5	1.5	1.5	3	0.064 - 8	20.0
Sheen -							
ceftazidime	103	2	1.5	2	8	0.75 – 16	98.1*
ciprofloxacin	123	0.19	0.19	0.19	0.5	0.047 – 1.5	93.5
tobramycin	123	1.5	1.5	1.5	3	0.047 – 5	30.6

*significant difference between Sheen + and Sheen – strains by chi-square, p=0.01.

### Sheen positive keratitis isolates are protease deficient

**Figure 2:**
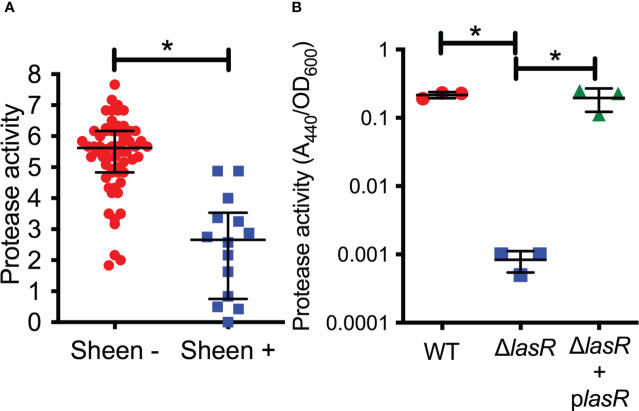
Secreted protease activity is reduced among sheen positive PA keratitis isolates and can be complemented. **(A)** Secreted protease activity by PA keratitis isolates. The zone of clearance (mm) on milk plate assay is shown. Each data point indicates the mean zone of clearance for an individual isolate. Medians and IQ ranges are shown. *, p<0.001 by Mann-Whitney. **(B)** Secreted protease activity measured by azocasein and normalized by bacterial density from sterile culture filtrates from the clinical isolate PAC (WT) and isogenic Δ*lasR* mutant and the mutant with wild-type *lasR* on a plasmid. The bacteria were grown in LB medium and were harvested at OD_600 =_ 2. Asterisks indicate p<0.01 between indicated groups by ANOVA with Tukey’s post-test, n=3, mean and SD are shown.

LasR is a known regulator of elastase B (*lasB*) and other proteases thought to aid the bacteria in microbial keratitis (Gambello and Iglewski, 1991). The protease activity of arbitrarily chosen keratitis isolates (14 sheen positive and 60 sheen negative) was assessed by measuring the zone of clearance on milk agar plates (**Figure 2A**). The sheen negative isolates had a zone of 5.6 mm versus 2.7 mm for the sheen positive isolate (p<0.0001, Mann-Whitney test).

To test this further, we generated a *lasR* deletion mutation in strain PaC (Kowalski et al., 2001) which is a fluoroquinolone resistant keratitis isolate. The PaC Δ*lasR* mutant was more than 100-fold reduced in protease activity compared to the wild type as measured using azocasein and this defect could be complemented by adding the wild-type *lasR* gene back on a plasmid (**Figure 2B**).

### The *lasR* alleles from sheen positive isolates were largely loss of function alleles

**Figure 3:**
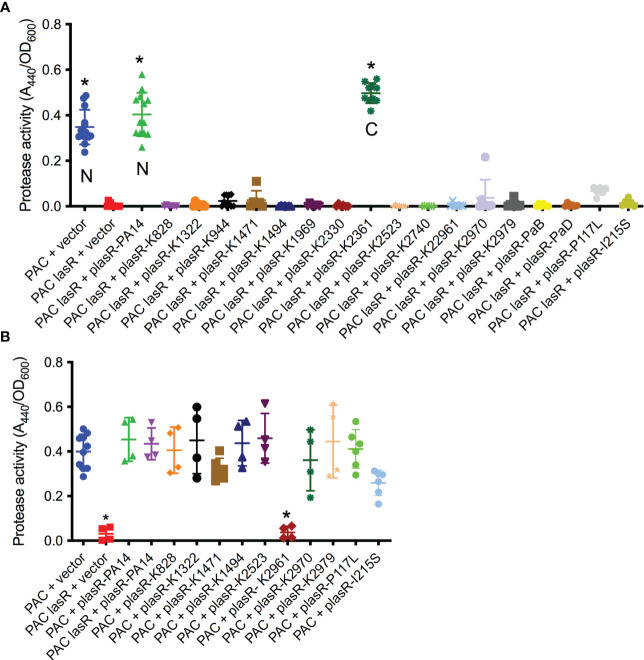
Tested *lasR* alleles from sheen positive isolates were loss of function and generally not dominant negative. **(A, B)** Protease activity in supernatants from overnight cultures (18-20h) grown in LB medium was measured using azocasein and normalized by culture density. n≥6, median and standard deviation is shown. **(A)** The vector alone negative control (pMQ132), wild-type *lasR* plasmid (plasR-PA14) positive control, and candidate plasmids were expressed in the wild-type PaC to establish base level or PaC *ΔlasR* to determine *lasR* function. “N” indicates a *lasR* allele from a sheen negative strain. “C” indicates *lasR* from a sheen positive isolate with no amino acid changes in the ORF transcript. Asterisk indicates p<0.001 by ANOVA with Tukey’s post-test. **(B)** As A, but plasmids were tested in the wild-type PaC to detect dominant negative activity. Asterisk indicates difference from PaC + vector by ANOVA, p<0.01.

While the sheen phenotype is linked to *lasR* loss of function mutations, the sheen phenotype can occur due to other mutations such as mutation of the *pqsH* gene which converts HHQ to PQS (Diggle et al., 2006; O'Connor et al., 2022). Several of the cloned *lasR* mutants were tested for function by expression in the PaC Δ*lasR* mutant followed by protease evaluation as a surrogate for LasR function (**Figure 3A**). Wild-type alleles from PA14 and K2361 restored wild-type levels of protease activity, whereas the *lasR* alleles cloned from sheen positive strains had highly reduced protease activity (**Figure 3A**).

Genes for the major two LasR variants I215S (14/22) and P117L (7/22) from the SCUT isolates were also cloned as above, moved into the PaC Δ*lasR* mutant and based on protease activity, both were loss of function mutations (**Figure 3A**). The PI117L mutant appeared to maintain some activity maintaining 16.6% of wild-type protease activity and 17-fold higher than the vector alone negative control, but was not significantly different than the vector alone negative control. The I215S allele allowed only 3.6-fold higher protease levels than the vector alone negative control.

The dominant negative status of the *lasR* alleles was also tested by expression in the wild-type PaC strain (**Figure 3B**). Notably, the K2961 allele strongly reduced protease activity in the PaC wild type, whereas the other tested *lasR* alleles did not significantly alter protease activity. The PaC strain with the *lasR*_K2961 allele expressed on a plasmid was remade to ensure that the effect was not artifactual and the reduced protease phenotype was again observed. A similar, although less severe reduction in protease activity was measured when the *lasR*_K2961 allele was expressed in the PA14 wild-type strain (**Figure 3B**). When the previously described *lasR* alleles from the India isolates were expressed at multicopy in the wild type bacteria, the P117L allele had no effect, but the I215S may have a dominant negative effect with a 43% reduction compared to the wild type expressing the PA14 *lasR* allele (**Figure 3B**).

### Clinical outcomes from keratitis isolates with and without sheen phenotype

Where possible the clinical outcomes of patients were determined from clinical records with n=49 for sheen negative isolates and n=19 for sheen positive. Of all of the evaluated variables only final average vision was significantly different (p=0.0106) (**Table 4**). Surprisingly, unlike the prior study evaluating *lasR* mutant associated keratitis (Hammond et al., 2016), the average visual outcomes were favorable for patients infected with the sheen positive isolates. Though not significantly different, the most severe outcomes, corneal transplants and enucleation, were absent in the sheen positive infected eyes, further suggesting reduced virulence by the sheen positive isolates.

**Table 4:** Clinical outcomes from keratitis patients.

	Sheen Negative (n=49)	Sheen Positive (n=19)	p-value
**Age**	56.4 ± 21.1 years	49.3 ± 23.6 years	p=0.2322
**Female**	73.5% (36/49)	42.1% (8/19)	**p=0.0152**
**Symptom duration**	3.5 ± 2.5 days (N=47)	3.3 ± 4.6 days (N=19)	p=0.8446
**Average Epi Defect Size**	26.7 ± 31.1 mm2 (N=44)	12.5 ± 21.2 mm2 (N=16)	p=0.0979
**Average Infiltrate Size**	25.0 ± 31.3 mm2 (N=44)	14.0 ± 21.0 mm2 (N=17)	p=0.1861
**Hypopyon present**	51.1% (24/47)	57.9% (11/19)	p=0.1180
**Average Initial VA**	1.70 ± 0.9 LogMAR	1.73 ± 0.9 LogMAR	p=0.9165
**Average Final VA**	1.1 ± 1.0 LogMAR	0.42 ± 0.56 LogMAR	**p=0.0106**
**Average time to resolution**	36.3 ± 38.2 days	34.8 ± 28.5 days	p=0.8835
**Management with Fluoroquinolone drops**	85.7% (42/49)	89.5% (17/19)	p=0.8824
**Management with Fortified drops**	83.7% (41/49)	73.7% (14/19)	p=0.6806
**Management with oral fluoroquinolone**	46.9% (23/49)	36.8% (7/19)	p=0.5744
**Management with topical steroids**	53.1% (26/49)	42.1% (8/19)	p=0.5664
**PKP**	14.3% (7/49)	0% (0/19)	p=0.1778
**Enucleation**	8.2% (4/49)	0% (0/19)	p=0.5702

VA, visual acuity regarding logMAR values (lower indicates better visual acuity).

PKP, penetrating keratoplasty (corneal transplant). Bold text indicates significant differences.

## Discussion

This study has demonstrated an increase in sheen positive *P. aeruginosa* keratitis isolates in a tertiary care hospital in the Eastern United States. This suggests that the abundance of sheen positive isolates is a general rather than a regional phenomenon. The reason for the increase observed in this study was not clear. The clinical microbiologist that collected the samples maintained the same collection protocol over the period of isolate collection, so differences in this would not account for the increase in sheen isolates.

Another consideration was whether the sheen positive isolates from our study are *lasR* mutants. The majority (22 out of 24) of sheen isolates that were sequenced had changes in the *lasR* sequence and those tested did not code for functional proteins. Because only the ORFs were cloned, other mutations that render the strain LasR-deficient could be missed, for example, promoter mutations. However, it is formally possible that in a small subset of the keratitis isolates, LasR-independent changes could be responsible for a subset of the sheen isolates. Therefore, we conclude that the majority if not all sheen positive keratitis isolates have defects in LasR function.

Whereas the sheen phenotype appears to be a good predictor of *lasR* mutant status, it has been observed that some *lasR* mutants do not manifest the sheen phenotype (Feltner et al., 2016; Mould et al., 2022). In this study the *lasR* gene was sequenced from three sheen negative isolates and all three had WT sequences; two were used to complement a Δ*lasR* strain for protease production and showed functional complementation. However, the protease levels from 7 out of 60 sheen negative isolates were close to the median for the sheen positive isolates suggesting that ~10% of the sheen negative isolates are defective in LasR function. Therefore, our data suggest that the sheen negative phenotype may have around a 10% error rate for predicting a functional LasR protein.

The Hammond study indicated worse visual outcomes for patients with sheen positive isolates (Hammond et al., 2016), by contrast patients in this study had strikingly better visual acuity, as well as no incidence of the severe outcomes of enucleation and corneal transplantation that were present in the sheen negative infected patients. The reason for this discrepancy is not clear, but could possibly be due to the different strains or the specific mutations associated with strains isolated in the SCUT study. Moreover, the SCUT study used a standard protocol for timing and methodology for obtaining visual acuity measurements, which lends more weight to that analysis.

Nevertheless, the reduced severity observed in the clinical data from this study were consistent with a recent paper using a rabbit corneal infection model that demonstrated reduced corneal perforation and bacterial proliferation of a *lasR* deletion mutant of strain PA14 compared to an isogenic wild type suggesting that LasR promotes keratitis severity (Romanowski et al., 2023). Studies with mice show mixed results with strain PA01 with *lasR* deletion mutations. The Pier group reported that C3H/HeN mice with scarified corneas required fewer *lasR* mutant bacteria to cause keratitis compared to the wild type (Preston et al., 1997). Whereas the Willcox group used the same bacterial strains with BALB/c mice and found indistinguishable infection frequencies for both bacterial strains, but reported that bacterial proliferation and severity scores were reduced in eyes infected with the *lasR* mutant (Preston et al., 1997).

Protease activity was used in this study as a reporter for LasR function; however, the reduction of protease production by the *lasR* mutants may contribute to reduced virulence. Several studies have linked extracellular proteases to the ability of *P. aeruginosa* to spread and proliferate in the cornea and to alter the immune response (Matsumoto, 2004; Willcox, 2007; O'Callaghan et al., 2019; Hilliam et al., 2020). For example, elastase injected into rabbit corneas causes corneal melting that can be prevented by elastase inhibitors (Pallares et al., 2021), though proteases are not absolutely necessary for *P. aeruginosa* to cause corneal inflammation (Hobden, 2002).

In conclusion, *lasR* mutant *P. aeruginosa* appear to be increasing among keratitis patients and this may be a world-wide phenomenon. The highly variable nature of the *lasR* mutations among isolates in our study does not unequivocally indicate whether the strains mutated during infection or prior to infection in general. The exception being identification of multiple isolates with a mutation at base pair 126 which suggests the existence of a regional endemic strain which is in agreement with a study by Hammond, et al (Hammond et al., 2016), which postulated that strains with existing *lasR* mutations initiate ocular infections. Interestingly, the identical insertion element in the same location was reported in the *lasR* gene from a *P. aeruginosa* isolated from a bean plant in Spain suggesting an environmental source (Ruiz-Roldan et al., 2021). Insertion elements interrupting the *lasR* ORF have also been observed among sheen positive isolates from young cystic fibrosis patients and other sources (Hoffman et al., 2009). Whether these *lasR* mutant strains are more or less virulent in keratitis is in question and more research is needed to determine the level of concern, however, our study suggests that the sheen positive *lasR* isolates are less pathogenic.

## Data availability statement

The original contributions presented in the study are included in the article/supplementary materials, further inquiries can be directed to the corresponding author.

## Ethics statement

The studies involving humans were approved by University of Pittsburgh Institutional Review Board. The studies were conducted in accordance with the local legislation and institutional requirements. Written informed consent for participation was not required from the participants or the participants’ legal guardians/next of kin in accordance with the national legislation and institutional requirements.

## Author contributions

RS: Data curation, Formal Analysis, Funding acquisition, Methodology, Project administration, Supervision, Validation, Writing – original draft, Writing – review & editing. SA: Data curation, Formal Analysis, Methodology, Writing – review & editing. NS: Formal Analysis, Investigation, Writing – review & editing. CS: Data curation, Formal Analysis, Investigation, Writing – review & editing. JR: Data curation, Investigation, Writing – review & editing. AG: Data curation, Formal Analysis, Investigation, Writing – review & editing. HS: Data curation, Formal Analysis, Investigation, Writing – review & editing. SM: Data curation, Formal Analysis, Investigation, Writing – review & editing. DD: Conceptualization, Methodology, Writing – review & editing. AM: Conceptualization, Investigation, Writing – review & editing. JC: Formal Analysis, Investigation, Methodology, Resources, Writing – review & editing. RC: Data curation, Formal Analysis, Methodology, Writing – review & editing. ER: Conceptualization, Formal Analysis, Investigation, Writing – review & editing. RK: Conceptualization, Funding acquisition, Resources, Writing – review & editing. MZ: Conceptualization, Formal Analysis, Methodology, Writing – original draft. VJ: Conceptualization, Data curation, Formal Analysis, Investigation, Writing – original draft.
